# ABA INSENSITIVE4 promotes rather than represses PHYA‐dependent seed germination in *Arabidopsis thaliana*

**DOI:** 10.1111/nph.16363

**Published:** 2019-12-30

**Authors:** Thiago Barros‐Galvão, Anuja Dave, Alison D. Gilday, David Harvey, Fabián E. Vaistij, Ian A. Graham

**Affiliations:** ^1^ Department of Biology Centre for Novel Agricultural Products University of York York YO10 5DD UK

**Keywords:** abscisic acid (ABA), ABSCISIC ACID INSENSITIVE4 (ABI4), far‐red light, germination, MOTHER‐OF‐FT‐AND‐TFL1 (MFT), PHYTOCHROME A (PHYA)

Light quality plays vital roles in the life cycle of plants. For example, in seeds of many species, light quality determines the levels of the gibberelic acid (GA) and abscisic acid (ABA) phytohormones, which promote and repress seed germination respectively (Seo *et al.*, 2006). In *Arabidopsis thaliana*, the photoreceptors phytochrome A (PHYA) and phytochrome B (PHYB) distinguish between full light (rich in red wavelength; R) and shade light (rich in far‐red wavelength; FR) to regulate seed germination (Lymperopoulos *et al.*, 2018). PHYB is reversibly activated and deactivated by R and FR light, respectively. Unlike the effect upon PHYB, both R and FR light irreversibly activate PHYA and once active, PHYA is more resistant to proteasome‐mediated degradation (Shinomura *et al.*, 1994, 1996; Debrieux & Fankhauser, 2010). Both PHYA and PHYB promote germination by targeting the transcription factor PHYTOCHROME INTERACTING FACTOR 1 (PIF1) for protein degradation (Shen *et al.*, 2005; Oh *et al.*, 2006). When PHYA and PHYB are inactive PIF1 accumulates and regulates expression of genes leading to low GA/ABA ratios, which in turn repress germination (Oh *et al.*, 2004, 2007; Kim *et al.*, 2016). Conversely, upon PHYA and PHYB activation, and the subsequent PIF1 degradation, GA/ABA ratios increase to promote germination.

In addition to their different light‐quality dependent activation, PHYA and PHYB have distinct patterns of accumulation: while PHYB accumulates from the beginning of seed imbibition; PHYA only accumulates after longer imbibition periods (Lee *et al.*, 2012). The fact that there are differences in response to light and accumulation patterns between PHYA and PHYB has been used to dissect their functions: short pulses of R and FR light at early stages of seed imbibition (before PHYA accumulates) are sufficient to reversibly activate and deactivate PHYB. Hence, two consecutive FR and R light pulses (FR/R; Fig. 1a) activate PHYB. By contrast, only one FR pulse (FR; Fig. 1a) deactivates PHYB. Exposure to FR light at later stages of imbibition activates PHYA, but still deactivates PHYB. Thus, an initial short FR light pulse followed later by a 60 min long FR light exposure (FR/FR; Fig. 1a), or a continuous 48 h FR light treatment (FR48; Fig. 1a), results in activation of PHYA and deactivation of PHYB.

**Figure 1:**
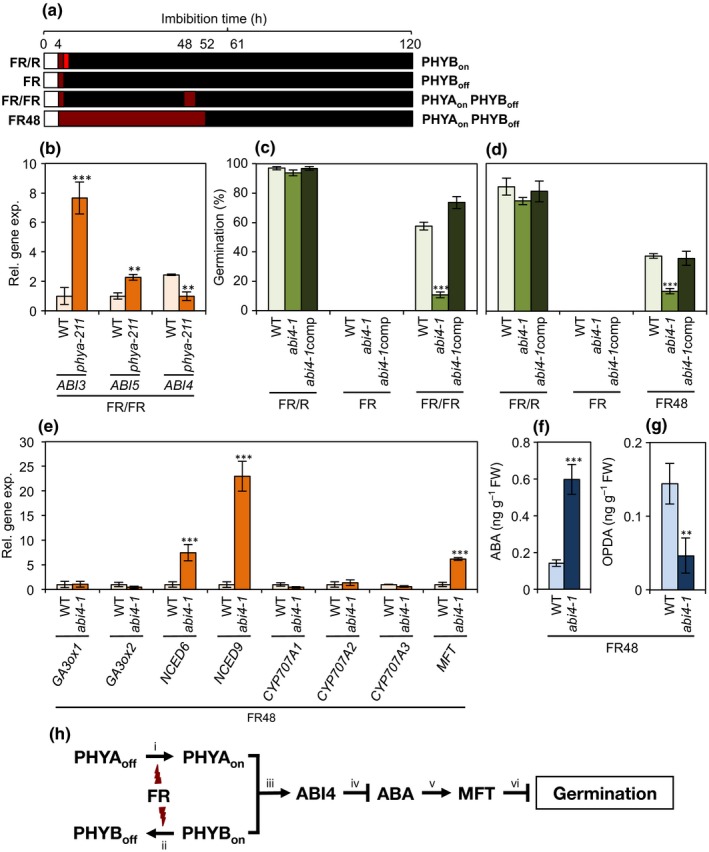
ABSCISIC ACID INSENSITIVE 4 (ABI4) promotes phytochrome A (PHYA)‐dependent germination and represses both abscisic acid (ABA) accumulation and MOTHER‐OF‐FT‐AND‐TFL1 (*MFT*) gene expression in *Arabidopsis thaliana*. (a) Schematic of the experimental set up. Seeds were imbibed on water‐agar plates for 4 h under low light and then treated with: (1) two successive 5 min far‐red (FR) and red (R) light pulses to activate PHYB (PHYB_on_) during the period when PHYA does not accumulate; (2) only one 5 min FR light pulse to deactivate PHYB (PHYB_off_) during the period when PHYA does not accumulate; (3) an initial 5 min FR light pulse followed 44 h later (48 h after imbibition, hai) by a second 60 min long FR light exposure to activate PHYA and deactivate PHYB (PHYA_on_ PHYB_off_); or (4) a continuous FR exposure for 48 h (FR48) to activate PHYA and deactivate PHYB (PHYA_on_ PHYB_off_). Seeds were kept in the dark between and after light treatments. (b) Relative expression of *ABI3*, *ABI5* and *ABI4* in FR/FR treated wild‐type (WT) and *phya‐211* seeds 12 h after the end of the second FR treatment (61 hai). (c, d) Germination (120 hai) of FR/R, FR and FR/FR (c) and FR/R, FR and FR48 (d) treated WT, *abi4‐1* and *abi4‐1*comp seeds. (e) Relative expression of *GA3ox1*, *GA3ox2*, *NCED6*, *NCED9*, *CYP707A1*, *CYP707A2* and *CYP707A3* in FR48 treated WT and *abi4‐1* seeds (52 hai). ABA (f) and 12‐oxo‐phytodienoic acid (OPDA) (g) levels in FR48 treated WT and *abi4*‐1 seeds (52 hai). Data are means ± SD of three (for gene expression) and four (for germination and phytohormones levels) biological replicates. Asterisks indicate statistically significant difference according to two‐tailed Student's *t*‐test (**, *P* < 0.01; ***, *P* < 0.001). (h) Model of the role of PHYA and ABI4 in the promotion of seed germination: FR light (FR/FR and FR48 treatments in our experimental set up) activates PHYA (PHYA_on_) (i) and deactivates PHYB (PHYB_off_) (ii). PHYA_on_ promotes *ABI4* gene expression (iii). ABI4 represses accumulation of ABA (iv) by inhibiting expression of the ABA‐biosynthesis *NCDE6* and *NCDE9* genes (not depicted in the model). ABA acts, at least partially, through MFT (v) to repress seed germination (vi).

ABA acts through the signalling factors ABA INSENSITIVE3 (ABI3), ABI4 and ABI5, which are B3‐, AP2‐ and bZIP‐type transcription factors, respectively (Finkelstein *et al.*, 1998; Finkelstein & Lynch, 2000; Clerkx *et al.*, 2003). These three factors were originally identified as mutations that resulted in seeds that were insensitive to ABA treatments (Koornneef *et al.*, 1984; Finkelstein, 1994). The corresponding genes were later found to also be involved in ABA signalling in other biological processes distinct from seed germination (Rohde *et al.*, 2000; Signora *et al.*, 2001; Rushton *et al.*, 2012). The roles of ABI3 and ABI5 in light‐dependent seed germination have been described previously: *ABI3* gene expression is induced under PHYB deactivating light conditions and, in turn, ABI3 controls expression of ABA‐response related genes including *ABI5* (Piskurewicz *et al.*, 2009). ABI5 plays more of a secondary role with a relatively modest effect under light conditions leading to PHYA activation and PHYB deactivation (Lee *et al.,* 2012). This is probably due to the fact that the jasmonic acid precursor oxylipin *cis*‐12‐oxo‐phytodienoic acid (OPDA) also plays a critical role in light‐quality dependent repression of seed germination in an ABI5 independent manner (Barros‐Galvão *et al.*, 2019).

Regarding ABI4, it has been shown to control lipid metabolism in seeds, sugar‐directed growth arrest, lateral root development, and plastid‐to‐nucleus retrograde signalling (Wind *et al.*, 2013). Intriguingly, previously published transcriptomic analysis revealed that, opposite to expectation, *ABI4* gene expression is repressed by FR light conditions, which are known to increase ABA levels (Oh *et al.*, 2009; Vaistij *et al.*, 2018). These observations prompted us to investigate the role of ABI4 on the light‐quality dependent germination pathway. We first compared *ABI3*,* ABI4* and *ABI5* gene expression upon FR/FR light treatment in *phya‐211* mutant seeds, which do not germinate under these conditions (Lee *et al.*, 2012). Twelve hours after the end of the second FR light treatment (61 h after imbibition, hai; Fig. 1a) *ABI3* and *ABI5* expression was, as expected, increased in mutant seeds compared to wild‐type control seeds (Fig. 1b). By contrast, *ABI4* expression was repressed in *phya‐211* seeds (Fig. 1b). This shows that PHYA, an inducer of germination, promotes *ABI4* expression. We then assessed germination of lack‐of‐function *abi4‐1* mutant seeds (120 hai; Fig. 1a). We also analysed seeds of an *abi4‐1* complemented line (*abi4‐1*comp). As expected, under FR/R conditions, seeds of all genetic backgrounds analysed germinated at similar high rates (Fig. 1c,d). Under FR light conditions, germination of all seeds was severely repressed (Fig. 1c,d). By contrast, under FR/FR and FR48 light conditions, while wild‐type and *abi4‐1*comp seeds germinated at relatively high and similar levels, *abi4‐1* mutant seeds germinated at lower rates (Fig. 1c,d). These observations demonstrate that, in contrast to what has been reported for ABI5 (Lee *et al.*, 2012), ABI4 promotes PHYA‐dependent germination.

Previous studies showed that ABI4 and ABI5 are not only involved in ABA signalling, but they also positively feedback to regulate expression of genes leading to reduced GA/ABA ratios (Lee *et al.*, 2012; Shu *et al.* 2013, 2016). This led us to assess whether expression of key genes involved in GA‐biosynthesis (*GA3ox1* and *GA3ox3*), ABA‐biosynthesis (*NCED6* and *NCDE9*), and ABA‐breakdown (*CYP701A1*, *CYP701A2* and *CYP701A3*) are regulated by ABI4 under FR48 light conditions (52 hai; Fig. 1a). We found that, while expression of GA‐biosynthesis and ABA‐breakdown related genes were unchanged, ABA‐biosynthesis genes were up regulated in *abi4‐1* seeds (Fig. 1e). This prompted us to measure ABA levels in FR48 light treated seeds (52 hai; Fig. 1a). We found that, in accordance with the increased *NCED6* and *NCED9* expression, ABA was present at higher levels in *abi4‐1* compared to wild‐type seeds (Fig. 1f). These results show that, in contrast to its role in promoting ABA accumulation in post‐germination developmental stages (Shu *et al.*, 2016), ABI4 represses ABA accumulation during PHYA‐dependent promotion of seed germination.

We reported previously that OPDA is a key repressor of germination (Dave *et al.*, 2011, 2016). More recently we also showed that, under FR light conditions, and to a lesser extent under FR48 light conditions, OPDA acts in parallel to the action of ABA (Barros‐Galvão *et al.*, 2019) in repressing seed germination. Hence, we also measured OPDA and found that levels were decreased in *abi4‐1* seeds (Fig. 1g). In our previous study, we showed that the abnormally high germination of ABA and OPDA deficient mutant seeds is repressed by either ABA or OPDA treatments (Barros‐Galvão *et al.*, 2019). Presumably, in the present study the high ABA levels that lead to germination inhibition compensate for the low OPDA levels in *abi4‐1* seeds.

In two previous studies from our laboratory, we demonstrated that MOTHER‐OF‐FT‐AND‐TFL1 (MFT) is a repressor of seed germination, that it is a key factor of the ABA‐signalling pathway, and that *MFT* gene expression is promoted by shade light (Vaistij *et al.*, 2013, 2018). This prompted us to assess whether *MFT* expression is regulated by ABI4. We found that, under FR48 light conditions (52 hai; Fig. 1a), *MFT* expression was increased in *abi4‐1* seeds (Fig. 1e). This observation shows that, as for *NCDE6* and *NCDE9*, ABI4 inhibits *MFT* expression. Whether this repression is due to direct or indirect ABI4–*MFT*/*NCDEs* interactions remains to be determined.

In conclusion, previous studies have established that ABI3, ABI4 and ABI5 are ABA‐signalling effectors repressing germination (Koornneef *et al.*, 1984; Finkelstein, 1994). It has also been demonstrated that ABI3 and ABI5 act under shade light conditions to inhibit germination (Piskurewicz *et al.*, 2009; Lee *et al.* 2012). In the current study, we reveal an unexpected role for ABI4 in promoting, rather than repressing, PHYA‐induced germination (Fig. 1h): upon light activation, PHYA promotes *ABI4* gene expression (probably through the action of PIF1). In turn, ABI4 represses expression of *NCDE6* and *NCDE9*, which leads to a decrease in ABA levels in the seed. ABI4 also reduces *MFT* gene expression either directly or indirectly through its effect on ABA, which itself promotes *MFT* expression (Xi *et al.*, 2010). Interestingly, it has been reported that, as under PHYA activating light conditions (Fig. 1b), the pattern of expression of *ABI4* is opposite to that of *ABI3* and *ABI5* in both Arabidopsis seed dormancy cycling (Footitt *et al.*, 2011, 2014) and in *Aethionema arabicum* light‐dependent seed germination (Mérai *et al.*, 2019). This suggests that the germination‐promoting role of ABI4 is a more general phenomenon. The evolutionary processes resulting in ABI4 function switching from a repressor to a promoter of seed germination depending on the environmental conditions remain to be elucidated.

## Methods

The mutant *abi4‐1* and *phya‐211* lines were described previously (Finkelstein, 1994; Reed *et al.*, 1994). The *abi4‐1*comp line was obtained by transformation of the mutant line with a pGREEN derived binary vector (pGTI0242ΔGR) carrying the *ABI4* coding sequence under the control of the CaMV 35S promoter. All Arabidopsis lines used in this work are of the Columbia ecotype. Plant growth conditions, seed collection, germination assays, RNA extraction, primer sequences, quantitative polymerase chain reactions (qPCRs) conditions and phytohormones extractions were described previously (Dave *et al.*, 2016; Vaistij *et al.*, 2018; Barros‐Galvão *et al.*, 2019). For gene expression analysis, transcript levels as determined by qPCR were normalized to *UBQ11* expression and expressed relative to the lower of the expression levels in each of the wild‐type vs mutant comparisons.

## Author contributions

TB‐G, FEV and IAG planned and designed the research. TB‐G, AD, ADG, DH and FEV performed experiments and analysed data. TB‐G, FEV and IAG wrote the manuscript.

## References

[nph16363-bib-0001] Barros‐Galvão T , Vaistij FE , Cole A , Dave A , Harvey D , Langer S , Larson TR , Graham IA . 2019 *cis*‐12‐Oxo‐phytodienoic acid plays a key role in controlling germination of *Arabidopsis thaliana* seeds in the shade. Journal of Experimental Botany 70: 5919–5927. 31326997 10.1093/jxb/erz337 PMC6812700

[nph16363-bib-0002] Clerkx EJ , Vries HB , Ruys GJ , Groot SP , Koornneef M . 2003 Characterization of green seed, an enhancer of *abi3*‐1 in Arabidopsis that affects seed longevity. Plant Physiology 132: 1077–1084. 12805635 10.1104/pp.103.022715 PMC167045

[nph16363-bib-0003] Dave A , Hernández ML , He Z , Andriotis VM , Vaistij FE , Larson TR , Graham IA . 2011 12‐Oxo‐phytodienoic acid accumulation during seed development represses seed germination in Arabidopsis. Plant Cell 23: 583–599. 21335376 10.1105/tpc.110.081489 PMC3077774

[nph16363-bib-0004] Dave A , Vaistij FE , Gilday AD , Penfield SD , Graham IA . 2016 Regulation of *Arabidopsis thaliana* seed dormancy and germination by 12‐oxo‐phytodienoic acid. Journal of Experimental Botany 67: 2277–2284. 26873978 10.1093/jxb/erw028 PMC4809285

[nph16363-bib-0005] Debrieux D , Fankhauser C . 2010 Light‐induced degradation of phyA is promoted by transfer of the photoreceptor into the nucleus. Plant Molecular Biology 73: 687–695. 20473552 10.1007/s11103-010-9649-9

[nph16363-bib-0006] Finkelstein RR . 1994 Mutations at two new Arabidopsis ABA response loci are similar to the *abi3* mutations. The Plant Journal 5: 765–771.

[nph16363-bib-0007] Finkelstein RR , Lynch TJ . 2000 The Arabidopsis abscisic acid response gene *ABI5* encodes a basic leucine zipper transcription factor. Plant Cell 12: 599–609. 10760247 10.1105/tpc.12.4.599 PMC139856

[nph16363-bib-0008] Finkelstein RR , Wang ML , Lynch TJ , Rao S , Goodman HM . 1998 The Arabidopsis abscisic acid response locus ABI4 encodes an APETALA 2 domain protein. Plant Cell 10: 1043–1054. 9634591 10.1105/tpc.10.6.1043 PMC144030

[nph16363-bib-0010] Footitt S , Clay HA , Dent K , Finch‐Savage WE . 2014 Environment sensing in spring‐dispersed seeds of a winter annual Arabidopsis influences the regulation of dormancy to align germination potential with seasonal changes. New Phytologist 202: 929–939. 24444091 10.1111/nph.12694 PMC4235297

[nph16363-bib-0009] Footitt S , Douterelo‐Soler I , Clay H , Finch‐Savage WE . 2011 Dormancy cycling in Arabidopsis seeds is controlled by seasonally distinct hormone‐signaling pathways. Proceedings of the National Academy of Sciences, USA 108: 20236–20241. 10.1073/pnas.1116325108 PMC3250134 22128331

[nph16363-bib-0011] Kim J , Kang H , Park J , Kim W , Yoo J , Lee N , Kim J , Yoon TY , Choi G . 2016 PIF1‐interacting transcription factors and their binding sequence elements determine the *in vivo* targeting sites of PIF1. Plant Cell 28: 1388–1405. 27303023 10.1105/tpc.16.00125 PMC4944412

[nph16363-bib-0012] Koornneef M , Reuling G , Karssen CM . 1984 The isolation and characterization of abscisic acid‐insensitive mutants of *Arabidopsis thaliana* . Physiologia Plantarum 61: 377–383.

[nph16363-bib-0013] Lee KP , Piskurewicz U , Turečková V , Carat S , Chappuis R , Strnad M , Fankhauser C , Lopez‐Molina L . 2012 Spatially and genetically distinct control of seed germination by phytochromes A and B. Genes and Development 26: 1984–1996. 22948663 10.1101/gad.194266.112 PMC3435500

[nph16363-bib-0014] Lymperopoulos P , Msanne J , Rabara R . 2018 Phytochrome and phytohormones: working in tandem for plant growth and development. Frontiers in Plant Sciences 9: 1037. 10.3389/fpls.2018.01037 PMC6072860 30100912

[nph16363-bib-0015] Mérai Z , Graeber K , Wilhelmsson P , Ullrich KK , Arshad W , Grosche C , Tarkowská D , Turečková V , Strnad M , Rensing SA *et al* 2019 *Aethionema arabicum*: a novel model plant to study the light control of seed germination. Journal of Experimental Botany 70: 3313–3328. 30949700 10.1093/jxb/erz146 PMC6598081

[nph16363-bib-0016] Oh E , Kang H , Yamaguchi S , Park J , Lee D , Kamiya Y , Choi G . 2009 Genome‐wide analysis of genes targeted by PHYTOCHROME INTERACTING FACTOR 3‐LIKE5 during seed germination in Arabidopsis. Plant Cell 21: 403–419. 19244139 10.1105/tpc.108.064691 PMC2660632

[nph16363-bib-0017] Oh E , Kim J , Park E , Kim JI , Kang C , Choi G . 2004 PIL5, a phytochrome‐interacting basic helix‐loop‐helix protein, is a key negative regulator of seed germination in *Arabidopsis thaliana* . Plant Cell 16: 3045–3058. 15486102 10.1105/tpc.104.025163 PMC527197

[nph16363-bib-0018] Oh E , Yamaguchi S , Hu J , Yusuke J , Jung B , Paik I , Lee HS , Sun TP , Kamiya Y , Choi G . 2007 PIL5, a phytochrome‐interacting bHLH protein, regulates gibberellin responsiveness by binding directly to the *GAI* and *RGA* promoters in Arabidopsis seeds. Plant Cell 19: 1192–1208. 17449805 10.1105/tpc.107.050153 PMC1913757

[nph16363-bib-0019] Oh E , Yamaguchi S , Kamiya Y , Bae G , Chung WI , Choi G . 2006 Light activates the degradation of PIL5 protein to promote seed germination through gibberellin in Arabidopsis. The Plant Journal 47: 124–139. 16740147 10.1111/j.1365-313X.2006.02773.x

[nph16363-bib-0020] Piskurewicz U , Turecková V , Lacombe E , Lopez‐Molina L . 2009 Far‐red light inhibits germination through DELLA‐ dependent stimulation of ABA synthesis and ABI3 activity. EMBO Journal 28: 2259–2271. 19556968 10.1038/emboj.2009.170 PMC2726693

[nph16363-bib-0021] Reed JW , Nagatani A , Elich TD , Fagan M , Chory J . 1994 Phytochrome A and phytochrome B have overlapping but distinct functions in Arabidopsis development. Plant Physiology 104: 1139–1149. 12232154 10.1104/pp.104.4.1139 PMC159274

[nph16363-bib-0022] Rohde A , Kurup S , Holdsworth M . 2000 ABI3 emerges from the seed. Trends in Plant Science 5: 418–419. 11203275 10.1016/s1360-1385(00)01736-2

[nph16363-bib-0023] Rushton DL , Tripathi P , Rabara RC , Lin J , Ringler P , Boken AK , Langum TJ , Smidt L , Boomsma DD , Emme NJ *et al* 2012 WRKY transcription factors: key components in abscisic acid signalling. Plant Biotechnology Journal 1: 2–11. 10.1111/j.1467-7652.2011.00634.x 21696534

[nph16363-bib-0024] Seo M , Hanada A , Kuwahara A , Endo A , Okamoto M , Yamauchi Y , North H , Marion‐Poll A , Sun TP , Koshiba T *et al* 2006 Regulation of hormone metabolism in Arabidopsis seeds: phytochrome regulation of abscisic acid metabolism and abscisic acid regulation of gibberellin metabolism. The Plant Journal 48: 354–366. 17010113 10.1111/j.1365-313X.2006.02881.x

[nph16363-bib-0026] Shen H , Moon J , Huq E . 2005 PIF1 is regulated by light‐mediated degradation through the ubiquitin‐26S proteasome pathway to optimize seedling photomorphogenesis in Arabidopsis. The Plant Journal 44: 1023–1035. 16359394 10.1111/j.1365-313X.2005.02606.x

[nph16363-bib-0027] Shinomura T , Nagatani A , Chory J , Furuya M . 1994 The induction of seed germination in *Arabidopsis thaliana* is regulated principally by phytochrome B and secondarily by phytochrome A. Plant Physiology 104: 363–371. 12232088 10.1104/pp.104.2.363 PMC159207

[nph16363-bib-0028] Shinomura T , Nagatani A , Hanzawa H , Kubota M , Watanabe M , Furuya M . 1996 Action spectra for phytochrome A‐ and B‐specific photoinduction of seed germination in *Arabidopsis thaliana* . Proceedings of the National Academy of Sciences, USA 15: 8129–8133. 10.1073/pnas.93.15.8129 PMC38887 8755615

[nph16363-bib-0029] Shu K , Chen Q , Wu Y , Liu R , Zhang H , Wang P , Li Y , Wang S , Tang S , Liu C *et al* 2016 ABI4 mediates antagonistic effects of abscisic acid and gibberellins at transcript and protein levels. The Plant Journal 85: 348–361. 26708041 10.1111/tpj.13109

[nph16363-bib-0030] Shu K , Zhang H , Wang S , Chen M , Wu Y , Tang S , Liu C , Feng Y , Cao X , Xie Q . 2013 ABI4 regulates primary seed dormancy by regulating the biogenesis of abscisic acid and gibberellins in Arabidopsis. PLoS Genetics 9: e1003577. 23818868 10.1371/journal.pgen.1003577 PMC3688486

[nph16363-bib-0031] Signora L , Smet I , Foyer C , Zhang H . 2001 ABA plays a central role in mediating the regulatory effects of nitrate on root branching in *Arabidopsis* . The Plant Journal 28: 655–662. 11851911 10.1046/j.1365-313x.2001.01185.x

[nph16363-bib-0032] Vaistij FE , Barros‐Galvão T , Cole AF , Gilday AD , He Z , Li Y , Harvey D , Larson TR , Graham IA . 2018 MOTHER‐OF‐FT‐AND‐TFL1 represses seed germination under far‐red light by modulating phytohormone responses in *Arabidopsis thaliana* . Proceedings of the National Academy of Sciences, USA 115: 8442–8447. 10.1073/pnas.1806460115 PMC6099910 30061395

[nph16363-bib-0033] Vaistij FE , Gan Y , Penfield S , Gilday AD , Dave A , He Z , Josse EM , Choi G , Halliday KJ , Graham IA . 2013 Differential control of seed primary dormancy in Arabidopsis ecotypes by the transcription factor SPATULA. Proceedings of the National Academy of Sciences, USA 110: 10866–10871. 10.1073/pnas.1301647110 PMC3696787 23754415

[nph16363-bib-0034] Wind JJ , Peviani A , Snel B , Hanson J , Smeekens SC . 2013 ABI4: versatile activator and repressor. Trends Plant Sciences 18: 125–132. 10.1016/j.tplants.2012.10.004 23182343

[nph16363-bib-0035] Xi W , Liu C , Hou X , Yu H . 2010 MOTHER OF FT AND TFL1 regulates seed germination through a negative feedback loop modulating ABA signaling in Arabidopsis. Plant Cell 22: 1733–1748. 20551347 10.1105/tpc.109.073072 PMC2910974

